# Broadband Perfect Absorber in the Visible Range Based on Metasurface Composite Structures

**DOI:** 10.3390/ma15072612

**Published:** 2022-04-01

**Authors:** Ran Wang, Song Yue, Zhe Zhang, Yu Hou, Hongda Zhao, Shitian Qu, Man Li, Zichen Zhang

**Affiliations:** 1 Microelectronics Instruments and Equipments R&D Center, Institute of Microelectronics of Chinese Academy of Sciences, Beijing 100029, China; wangran@ime.ac.cn (R.W.); yuesong@ime.ac.cn (S.Y.); zhangzhe1@ime.ac.cn (Z.Z.); houyu@ime.ac.cn (Y.H.); zhaohongda@ime.ac.cn (H.Z.); qushitian21@mails.ucas.ac.cn (S.Q.); liman@ime.ac.cn (M.L.); 2 School of Microelectronics, University of Chinese Academy of Sciences, Beijing 101499, China

**Keywords:** broadband perfect absorber, metasurface composite structure, material intrinsic absorption, coupling resonance

## Abstract

The broadband perfect absorption of visible light is of great significance for solar cells and photodetectors. The realization of a two-dimensional broadband perfect absorber in the visible range poses a formidable challenge with regard to improving the integration of optical systems. In this paper, we numerically demonstrate a broadband perfect absorber in the visible range from 400 nm to 700 nm based on metasurface composite structures. Simulation results show that the average absorptance is ~95.7% due to the combination of the intrinsic absorption of the lossy metallic material (Au) and the coupling resonances of the multi-sized resonators. The proposed perfect absorber may find potential applications in photovoltaics and photodetection.

## 1. Introduction

A broadband perfect absorber in the visible range has great potential for solar energy harvesting [1,2,3] and photoelectric detection [4,5]. Naturally occurring materials, such as organic dyes and inorganic pigments, usually exhibit insufficient absorption in the visible range for many modern photonic applications [6]. Metamaterials are made up of artificially subwavelength nanostructures, providing numerous unconventional optical properties [7]. Their electromagnetic properties can be easily adjusted by the size and geometry of the nanostructures. Based on the impedance match between the designed metamaterial and the free space, the first metamaterial perfect absorber was demonstrated in 2008 in the microwave band [8]. Since then, the perfect absorber has been extended to the terahertz [9,10,11], mid-infrared [12], infrared [13,14,15], and visible range [16,17,18].

The bandwidth of an absorber is important for many scientific and technical applications. The narrowband absorbers are quite important for sensing applications, such as temperature or refractive index sensing, absorption filtering, and optical signal processing [19,20,21]. On the other hand, broadband absorbers facilitate seminal applications encompassing photovoltaic cells and optoelectronic detectors. The reported scheme for an broadband absorber based on metamaterials mainly relies on multiple vertically layered metamaterials [22,23,24]. However, due to the laminated structures, the reported absorbers are bulky and complex for use in fabrication. The realization of an ultrathin broadband perfect absorber is of great significance for integration applications. Metasurfaces comprise subwavelength constituent elements within an optically thin layer that can alter the amplitude, phase, and polarization of an incident electromagnetic wave [25,26]. The development of metasurfaces provides a unique opportunity for the planarization and miniaturization of the broadband perfect absorber [27,28]. Two-dimensional broadband perfect absorbers with a horizontal arrangement of several resonators in different sizes were obtained [29,30]. However, the absorption spectrum usually consists of several discrete peaks and the average absorption achieved is not high enough. Additionally, the use of a perfect absorber with a continuous broad bandwidth in the visible range is more favorable for many practical applications.

In this paper, a broadband perfect absorber based on metasurface composite structures in the visible range is demonstrated. The simulation results illustrate that the perfect absorber yields a ~95.7% average absorptance in the visible range (400–700 nm). The perfect absorption mechanism lies in the combination of the intrinsic absorption of the gold material (400 nm–550 nm) and the coupling resonances of multi-sized resonators (550 nm–700 nm) according to the simulation. Given the broad absorption bandwidth, high absorptance, and ultrathin structure, the designed metasurface absorbers may find potential applications in solar cells [31,32] and photodetection [33,34].

## 2. Simulation and Discussion

**Figure 1:**
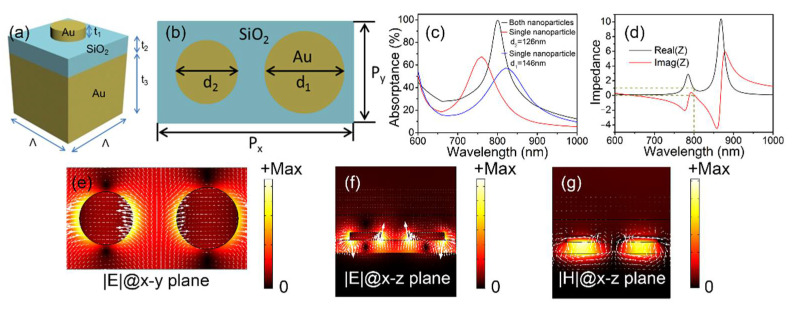
(**a**) The considered metal–insulator–metal unit cell. (**b**) The metasurface composite structures. (**c**) The simulated absorption spectra of the metasurface composite structures with both nanoparticles (black solid curve) and with a single nanoparticle (red solid curve for d_2_ = 126 nm and blue solid curve for d_1_ = 146 nm) under x polarization. (**d**) The impedance spectra (real and imaginary parts). The electric field distributions in (**e**) the x–y plane and (**f**) the x–z plane. (**g**) The magnetic field (color plot) and electric current (white arrows) distributions.

As plotted in Figure 1a, the unit cell of a metasurface is a metal–insulator–metal (MIM) sandwich structure. This consists of a circular-shaped gold nanoparticle and a gold substrate separated by a silicon dioxide (SiO_2_) spacer. The thicknesses of nanoparticles, SiO_2_ layer, and gold substrate are t_1_ = 30 nm, t_2_ = 50 nm, and t_3_ = 100 nm, respectively. The lateral dimension is Λ = 240 nm. The metasurface composite structures consist of two differently sized nanoparticles with diameters of d_1_ = 146 nm and d_2_ = 126 nm. Figure 1b plots the top view of the metasurface composite structures with periods of P_x_ = 2Λ = 480 nm and P_y_ = Λ = 240 nm. The absorption characteristics of the metasurface composite structures are simulated using the COMSOL Multiphysics V5.4 (2018, Stockholm, Sweden) with periodic boundary conditions. The refractive index of the SiO_2_ is 1.45 and the permittivity of gold as a function of the incident wavelength is taken from the experimental results [35]. The perfect absorption is obtained based on the parameter optimization of the diameters of the gold nanoparticles. The simulated absorption spectra of the metasurface composite structures (with both nanoparticles, black solid curve) and the absorption spectra of each constituent nanoparticle (red solid curve for d_2_ = 126 nm and blue solid curve for d_1_ = 146 nm) are plotted in Figure 1c under x polarization. For the metasurface composite structures with both nanoparticles, the absorptance is ~99.64% and the bandwidth Δλ_FWHM_ (full width at half maximum) is 67 nm at the resonant wavelength of λ_res_ = 800 nm. For the metasurface structures with a single nanoparticle of d_2_ = 126 nm, the absorptance is ~67.16% and Δλ_FWHM_ = 98 nm at λ_res_ = 760 nm. For the metasurface structures with a single nanoparticle of d_1_ = 146 nm, the absorptance is ~57.18% and Δλ_FWHM_ = 131 nm at λ_res_ = 820 nm.

The absorptance $A\left(\lambda \right)$ is obtained from $A\left(\lambda \right)=1-T\left(\lambda \right)-R\left(\lambda \right)$. $T\left(\lambda \right)$ is transmittance, while $R\left(\lambda \right)$ is reflectance. The transmittance $T\left(\lambda \right)$ is zero since the thickness of the gold substrate is t_3_ = 100 nm, which is greater than the skin depth of the gold film. As a result, $A\left(\lambda \right)=1-R\left(\lambda \right)$. The reflectance can be minimized until zero under the condition of impedance matching, resulting in the perfect absorption of $A\left(\lambda \right)\sim 100\;\%$. The impedance *Z* of the metasurface composite structures can be calculated according to the following equation [36]:

(1) $$Z=\sqrt{\frac{1+S_{11}{}^{2}-S_{21}^{2}}{1-S_{11}{}^{2}-S_{21}^{2}}}$$

Here, *S*_11_ is the complex reflection coefficient while *S*_21_ denotes the complex transmission coefficient. Due to the optical thickness of the gold substrate, *S*_21_ is zero. The simulated real and imaginary parts of the impedance are presented in Figure 1d. The real part is 1 and the imaginary part is 0 at the resonant wavelength of 800 nm; both of these are perfectly matched to the vacuum values and result in a perfect absorption effect.

To illuminate the absorption mechanism, the norm of the electric field (|***E***|, color plot) and the local electric field distribution at each position (white arrows) in the x–y plane through the nanoparticles 15 nm above the SiO_2_ layer and the x–z plane are plotted in Figure 1e,f under x polarization at the resonant wavelength of 800 nm. |***E***| shows that strong coupling exists between the two nanoparticles, and the absorption spectrum of the metasurface composite structures with both nanoparticles can be viewed as a coupling superposition of the absorption spectrum of each constituent nanoparticle. The white arrows indicate that the electric dipole resonance is excited in the nanoparticles when an x-polarized incident beam normally reaches the metasurface composite structures. Meanwhile, the norms of the magnetic field (|***H***|, color plot) and the electric current (white arrows) of the metasurface composite structures in the x–z plane are shown in Figure 1g. The antiparallel electric currents are induced in both the gold nanoparticles and substrate, and a strong magnetic dipole resonance is generated in the thin SiO_2_ layer [37]. As a result, impedance matching and the perfect absorption effect can be obtained at the resonance wavelength [38].

**Figure 2:**
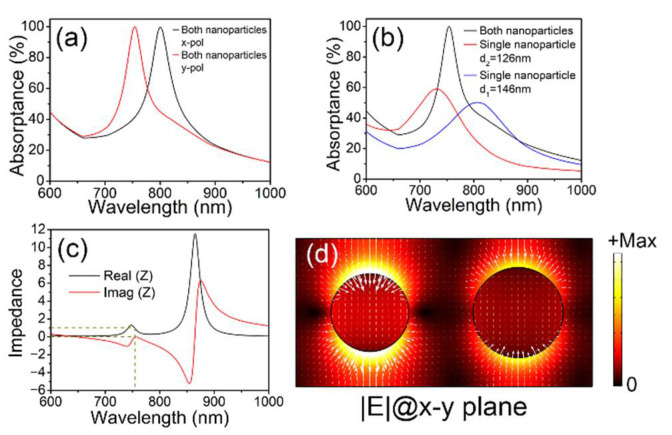
(**a**) The simulated absorption spectra of the metasurface composite structures under x and y polarizations. (**b**) The simulated absorption spectra of the metasurface composite structures with both nanoparticles (black solid curve) and with a single nanoparticle (red solid curve for d_2_ = 126 nm and blue solid curve for d_1_ = 146 nm) under y polarization. (**c**) The impedance spectra (real part and imaginary parts). (**d**) The electric field distributions.

Figure 2a plots the simulated absorption spectra of the metasurface composite structures under x and y polarizations with the resonant wavelengths of 800 nm and 754 nm, respectively. For y-polarized incidence, the absorption spectra of the metasurface composite structures with both nanoparticles (black solid curve) and with each constituent nanoparticle (red solid curve for d_2_ = 126 nm and blue solid curve for d_1_ = 146 nm) are simulated in Figure 2b. For the metasurface composite structures with both nanoparticles, the absorptance is ~99.95% and Δλ_FWHM_ = 64 nm at λ_res_ = 754 nm. The absorptance is ~59.08% and Δλ_FWHM_ = 153 nm at λ_res_ = 731 nm for the metasurface structures with a single nanoparticle of d_2_ = 126 nm. As for the metasurface structures with a single nanoparticle of d_1_ = 146 nm, the absorptance is ~50.23% and Δλ_FWHM_ = 170 nm at λ_res_ = 806 nm. The impedance is simulated in Figure 2c. The real and imaginary parts are perfectly matched to the vacuum values at λ_res_ = 754 nm. Figure 2d plots the norm of the electric field (|***E***|, color plot) and local electric field distribution (white arrows) in the x–y plane through the nanoparticles 15 nm above the SiO_2_ layer at λ_res_ = 754 nm. The electric dipole resonance can be easily observed in the nanoparticles. It is found that there is no strong coupling between the two nanoparticles, and the absorption spectrum of the metasurface composite structures with both nanoparticles can be viewed as a simple superposition of the absorption spectrum of each constituent nanoparticle.

**Figure 3:**
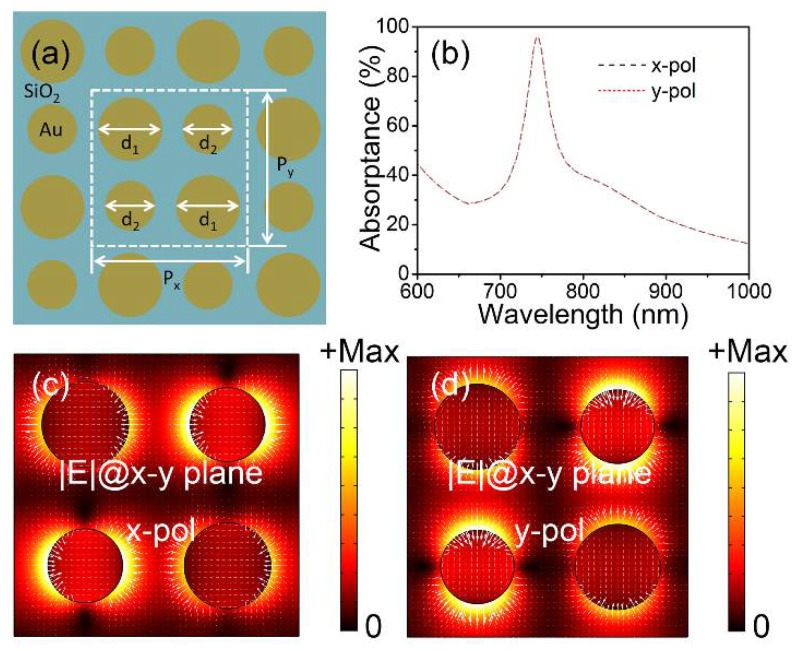
(**a**) The metasurface composite structures with four diagonal symmetric gold nanoparticles. (**b**) The simulated absorption spectra under x and y polarization. The electric field distributions under (**c**) x and (**d**) y polarization at λ_res_ = 745 nm.

The comparison between Figure 1c and Figure 2a shows that the perfect absorption characteristics are polarization-dependent because of the asymmetry of the geometry along the x- and y-axes. To improve the polarization dependence, the metasurface composite structures are designed with four diagonal symmetric gold nanoparticles above the SiO_2_ layer, as shown in Figure 3a. The geometrical parameters are d_1_ = 146 nm, d_2_ = 126 nm, and P_x_ = P_y_ = 2Λ = 480 nm, respectively. The thicknesses of the nanoparticles (t_1_), SiO_2_ layer (t_2_), and gold substrate (t_3_) are the same as those in Figure 1a. The simulated polarization-insensitive absorption spectra are presented in Figure 3b under x (black dashed curve) and y (red dotted curve) polarization. The absorptance is ~95.88% and the Δλ_FWHM_ is 55 nm at λ_res_ = 745 nm. The norms of the electric field (|***E***|, color plot) and local electric field distributions (white arrows) of the metasurface composite structures in the x–y plane are plotted in Figure 3c,d under x polarization and y polarization at λ_res_ = 745 nm. The electric dipole resonances can be separately observed along the x-axis and y-axis. It can be seen that, due to the symmetry of the composite structure, the absorption spectra under x- and y-polarization are identical, indicating the polarization insensitivity of the composite structure.

**Figure 4:**
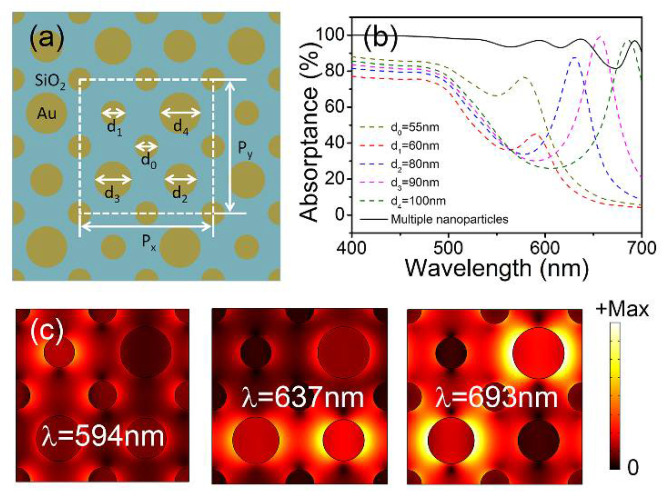
(**a**) The metasurface composite structures with five differently sized nanoparticles. (**b**) The simulated absorption spectra of the metasurface comprising each constituent gold nanoparticle and the metasurface composite structures with multiple nanoparticles under x-polarized incident light. (**c**) The electric field distributions (|***E***|) at λ_res_ = 594 nm, 637 nm, and 693 nm.

To achieve the perfect absorption in a broader wavelength range, five differently sized nanoparticles were designed in the metasurface composite structures, as plotted in Figure 4a. The diameters of the gold nanoparticles were optimized as d_0_ = 55 nm, d_1_ = 60 nm, d_2_ = 80 nm, d_3_ =90 nm, and d_4_ = 100 nm, respectively. The thicknesses of the nanoparticles, SiO_2_ layer, and gold substrate were t_1_ = 30 nm, t_2_ = 20 nm, and t_3_ = 100 nm, respectively. The subwavelength lateral dimension was reduced to Λ =160 nm to minimize the influence of the diffraction effects. As a result, the period was P_x_ = P_y_ = 2Λ = 320 nm. The absorption spectra were simulated (as presented in Figure 4b) under x-polarized incident light (black solid curve). It can be observed that the absorptance was beyond 90% in the wavelength ranges of 400–650 nm and 685–700 nm. For the broad visible wavelength range of 400–700 nm, the average absorptance was ~95.7%. Additionally, to illustrate the perfect absorption mechanism, the absorption spectra of the metasurface comprising each constituent gold nanoparticle were simulated (as plotted in Figure 4b) with diameters of 55, 60, 80, 90, and 100 nm. This was achieved by setting only one nanoparticle as a Au nanoparticle and all other nanoparticles as air in each simulation; this procedure was repeated for all constituent nanoparticles. The spectra exhibited pronounced resonance peaks at wavelengths of 580, 590, 630, 657, and 685 nm with absorptance values of 76.6%, 45.0%, 87.9%, 99.2%, and 97.3%, respectively. The resonant wavelengths were redshifted with the increase in the diameters of the nanoparticles. The power loss accumulated at the resonant wavelengths, where the energy of the incident light was converted into heat, resulting in a high level of absorption. As the designed five adjacent resonance peaks were so close, they could couple with each other and merge into a broad absorption band (550–700 nm). Meanwhile, the absorption in the shorter wavelength (400–550 nm) originated from the intrinsic absorption of the gold material, with an absorptance close to unity. The finite-element simulations shown in Figure 4c further verify the broadband perfect absorption mechanism in the range of 550–700 nm. The norm of the electric field (|***E***|, color plot) was plotted in the x–y plane through the nanoparticles 15 nm above the SiO_2_ layer under x-polarized incident lights. The coupled electric dipole resonances were observed at the different resonance peaks of 594 nm, 637 nm, and 693 nm according to the absorption spectra of the metasurface composite structures with multiple nanoparticles (black solid curve in Figure 4a). It can be observed that the broadband absorption in the range of 550–700 nm originated from the superposition of the coupling resonances of the multi-sized nanoparticles.

**Figure 5:**
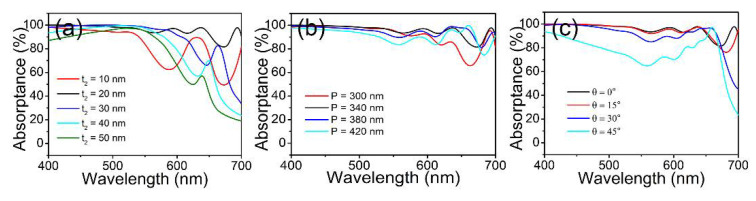
The simulated absorption spectra of the metasurface composite structures as functions of (**a**) the thickness of the SiO_2_ layer t_2_, (**b**) the period *P*, and (**c**) the incident angle θ under x polarization.

Subsequently, the influence of geometrical parameters on the proposed perfect absorber was investigated. Figure 5a plots the absorption spectra with respect to the thickness of the SiO_2_ layer t_2_. The average absorptance was 82.8%, 95.7%, 88.2%, 80.2%, and 73.2% when t_2_ = 10 nm, 20 nm, 30 nm, 40 nm, and 50 nm, respectively. On the other hand, the absorption spectra are presented in Figure 5b as functions of the period *P*. The average absorptance was 91.5%, 95.7%, 94.7%, and 91.3% when P = 300 nm, 340 nm, 380 nm, and 420 nm, respectively. It can be seen that the optimized perfect absorption was obtained when t_2_ = 20 nm and P = 340 nm, which is in accordance with the geometrical parameters shown in Figure 4. In addition, the absorption dependence at oblique incidence is illustrated in Figure 5c. Compared with the normal incidence (θ = 0°), the average absorptance decreased to 94.9%, 90.3%, and 75.6% when θ = 15°, 30°, and 45°, respectively. It can be observed that the broadband perfect absorption performance could be maintained when the incident angle increased up to 30°.

The anticipated fabrication of the proposed perfect absorber was based on the standard electron beam lithography steps [39]. Firstly, gold film with a thickness of 100 nm was deposited on a silicon substrate using electron beam evaporation. Secondly, a silicon dioxide film with an optimized thickness was formed by chemical vapor deposition. Finally, an array of multi-sized gold nanoparticles was fabricated using electron beam lithography, metal deposition, and a lift-off process. Based on high-resolution electron beam lithography (EBL) with a negative-tone hydrogen silsesquioxane (HSQ) resist and the lift-off process [40,41], gold nanoparticles with different-sized diameters could be reliably fabricated to satisfy the designed structural parameters (e.g., d_0_ = 55 nm, d_1_ = 60 nm, d_2_ = 80 nm, d_3_ =90 nm, and d_4_ = 100 nm). Additionally, the absorption mechanism (the material intrinsic absorption and the coupling resonances of the multi-sized resonators) allows for a relaxed tolerance of the nanoparticle diameters in practical fabrication on the condition that the diameters are evenly distributed within a certain range (from 55 nm to 100 nm). Slight deviations from the original diameter may lead to a shift in a single absorption peak, but the broadband absorption effect will remain.

## 3. Conclusions

In conclusion, in this research a broadband perfect absorber was proposed and numerically studied in the visible range based on metasurface composite structures. The simulated average absorptance was ~95.7% under x polarization in the wavelength range of 400–700 nm. The intrinsic absorption of gold material contributed to the absorption in the range of 400 to 550 nm, and the superposition of multiple resonances of composite nanoparticles contributed to the absorption in the range of 550 to 700 nm. A systematic strategy for the structure of broadband perfect absorbers was thus established. Based on the geometry scalability, the perfect absorber may operate at other wavelength ranges. Given their broad spectral absorption range and high absorptance, the designed metasurface absorbers may find potential applications in enhancing photovoltaics and photodetection.

## Data Availability

Not applicable.
